# Reproductive system and agronomic performance of the wild husk tomatillo *Physalis gracilis*

**DOI:** 10.1186/s40529-026-00500-7

**Published:** 2026-10-14

**Authors:** Omar Enríquez-Antonio, María del Pilar Zamora-Tavares, Lislie Solís-Montero, Fabián A. Rodríguez-Zaragoza, Carla Vanessa Sánchez-Hernández, Gabriela Alcalá-Gómez, José Alberto Sánchez-Nuño, Ofelia Vargas-Ponce

**Affiliations:** 1 Centro Universitario de Ciencias Biológicas y Agropecuarias, Departamento Botánica y Zoología, Maestría en Biosistemática y Manejo de Recursos Naturales y Agrícolas, Instituto de Botánica, , Universidad de Guadalajara, Camino Ing. Ramón Padilla Sánchez 2100, Las Agujas, Zapopan, Jalisco 45200 México; 2 Centro Universitario de Ciencias Biológicas y Agropecuarias, Laboratorio Nacional de Identificación y Caracterización Vegetal (LaniVeg), Departamento Botánica y Zoología, Instituto de Botánica, Universidad de Guadalajara, Camino Ing. Ramón Padilla Sánchez 2100, Las Agujas, Zapopan, Jalisco CP 45200 México; 3 Grupo Académico Biología y Ecología de Artrópodos Benéficos, Departamento Ecología de Artrópodos y Manejo de Plagas, El Colegio de la Frontera Sur, Carretera Antiguo Aeropuerto km 2.5, Tapachula, CP 30700 México; 4 Centro Universitario de Ciencias Biológicas y Agropecuarias, Laboratorio de Ecología, Departamento Ecología Aplicada, Universidad de Guadalajara, Conservación y Taxonomía (LEMITAX), Camino Ing. Ramón Padilla Sánchez 2100, Las Agujas, Zapopan, Jalisco CP 45200 México; 5 Centro Universitario de Ciencias Biológicas y Agropecuarias, Depto. Producción agrícola, Universidad de Guadalajara, Camino Ing. Ramón Padilla Sánchez 2100, Las Agujas, Zapopan, Jalisco CP 45200 México; 6 Secretaría de Ciencias, Tecnología e Innovación (SECIHTI), Av. Insurgentes Sur 1582, Colonia Crédito Constructor, Alcaldía Benito Juárez, Humanidades, México City, CP 03940 México

**Keywords:** Cultivation, Novel fruits, Underutilized *Physalis* species, Plant reproduction, Solanaceae

## Abstract

*Physalis gracilis* is a wild perennial herb with sweet, orange-colored fruits harvested for household consumption in Mexico. For species with potential for cultivation and nutritional value, a basic information is known their reproductive system. This study aimed to evaluate the reproductive system of *P. gracilis* and its agronomic performance. Floral longevity and development stages were determined in 30 flower buds until floral senescence, using individuals grown under greenhouse and open-field conditions. Six artificial cross treatments were conducted to characterize the reproductive system, and self-incompatibility and pollen limitation indices were calculated. Stigma receptivity was assessed using hydrogen peroxide. To assess agronomic performance, an open-field crop was established within three accessions, each with 48 individuals (in total 144 plants); six descriptors were evaluated from five fruits per plant per descriptor. Artificial crossing showed that the three accessions exhibited a completely self-incompatible reproductive system; however, it was not pollen limitated. *P. gracilis* responded positively to agricultural management (93.8% plant survival) and showed a potential average yield of 8.178 t·ha^− 1^. Equatorial diameter of the fruit ranged from 11 to 22 mm. The average sweetness was 11.29° Brix, and the pH was 4.18. This study demonstrates that *P. gracilis* could be a candidate as a new open-field crop with desirable fruit traits, such as weight, size, and sweetness.

## Introduction

Globally, wild plants are important for human nutrition (Bharucha and Pretty 2010). In Mexico, approximately 5,000 non-domesticated plant species are used in their wild state or as tolerated species within agroecosystems and traditional gardens (Casas et al. 1996). There is increasing interest in characterizing wild species with food potential to identify their nutritional attributes and develop novel crops (Prohens et al. 2003). The Solanaceae family includes species of major horticultural relevance, such as potato (*Solanum tuberosum* L.), tomato (*Solanum lycopersicum* L.), chili pepper (*Capsicum* spp.), and husk tomato (*Physalis* spp.) (Martínez et al. 2017), along with several wild relatives that could enhance food agrobiodiversity.

The reproductive system of plants species can be self-compatible, self-incompatible or a mixed system (Goodwillie et al. 2005). A key factor in species of agronomic interest is known their reproductive system and how pollination occurs whether through external pollen (cross-pollination) or the plant’s own pollen (self-pollination) leading to fertilization and the formation of fruits and seeds (Klein et al. 2007; Muñoz et al. 2020). This information is important for establishing crops in open fields or greenhouses. Most crops (75%) are self-compatible plants (SC), potentially self-pollinated with their own pollen, which rapidly selects and achieves cultivar uniformity of a desirable phenotype but with costs of inbreeding (Bixby and Levin 1996; Dempewolf et al. 2012; Rick 1988a, b). In contrast, few species are completely or partially self-incompatible (SI) (SI; they present a mechanism to reject their own pollen). In fact, the Solanaceae family presents an RNA-ase gametophytic self-incompatibility system (i.e., haploid gametophytes determine the mechanism of recognition and rejection of self-pollen based on RNases; Igic and Kohn 2001). In particular, *Physalis* species are predominantly allogamous (Menzel 1951). However, recent studies on the reproductive biology of *Physalis*, conducted mainly on wild species or those under incipient utilization, have revealed variations in their reproductive systems. For example, in *P. philadelphica*, artificial selection through cultivation has favored self-incompatibility in domesticated populations; whereas, in wild populations, self-pollination seems advantageous, as it produces seeds that germinate faster than those of cultivated plants (Solís-Montero et al. 2021). In *P. acutifolia*, self-incompatibility is predominant, although intraspecific variation has been observed, with some populations exhibiting self-pollination (Pretzs and Smith 2021). Meanwhile, *P. angulata*, *P. pubescens*, *P. micrantha* Link, and *P. nicandroides* reproduce mainly through self-pollination (Azeez 2020; Figueiredo et al. 2020; Ojeda-Flores 2024), and *P. peruviana* exhibits a mixed system (Ramírez et al. 2021). Whereas *P. viscosa* L. and *P. cinerascens* (Dunal) Hitchc. are allogamous (Sullivan 1985; Ojeda-Flores 2024).

The genus *Physalis* includes several species that are economically and nutritionally important (Puente et al. 2011; Martínez et al. 2023, Shenstone et al. 2020). The edible fruits, which are berries with an acidic or sweet taste, possess nutraceutical properties (Herrera et al. 2011). In the Americas, two species of pre-Hispanic crops of national significance are *Physalis peruviana* L. (uchuva, aguaymanto, cape gooseberry) in South America (Puente et al. 2011; Herrera et al. 2011) and *Physalis philadelphica* Lam. (husk tomato) in North America, mainly in Mexico (Zamora-Tavares et al. 2015; Alcalá-Gómez et al. 2022). Because of its sweetness, cape gooseberry fruit is consumed fresh or processed and is an important export product (Novoa et al. 2006; Naranjo-Durán et al. 2023). Husk tomato fruits are used in dishes and sauces because of their acidity. Both species are produced outside their native range (Mulato-Brito and Peña Lomeli 2007; Panayotov and Popova 2014; Singh et al. 2014) and are internationally commercially. In contrast, other species of *Physalis* are cultivated locally for self-consumption or cultural use. For example, over the last 50 years, *P. angulata* L. (“tomatillo milpero”), a native species, has been cultivated in the central region of Jalisco, Mexico (Vargas-Ponce et al. 2016). Historically, the fruits of this species have been used as food and medicine in Brazil, Colombia, Peru, and Mexico (Rengifo and Vargas 2013; Vargas-Ponce et al. 2016). Likewise, the backyard cultivation of *P. grisea* (Waterf.) M. Martínez (goldenberry) and *P. longifolia* Nutt. for consumption of their sweet fruits is common in the United States of America (Kindscher et al. 2012). Recently, the agronomic potential of several wild and weedy populations of *P. angulata* has been confirmed (Morales-Saavedra et al. 2019). Also, the agronomic productivity of *P. acutifolia* (Miers) Sandw., *P. chenopodifolia* Lam., and *P. pubescens* L. have been demonstrated. These species hold cultural significance for certain ethnic groups in Northwest and Central Mexico (Kindscher et al. 2012; Valdivia-Mares et al. 2016). Similarly, some *Physalis* species have been evaluated as new crops outside their native range, including *P. pruinosa* L. and *P. nicandroides* Schltdl. (Singh et al. 2014).

*Physalis gracilis* Miers is a wild species with potential for agronomic development because their sweet fruits (“costomate”, “costomatillo”) are highly valued for family consumption and, on a very small scale, for sale in local markets in Mexico (Santiaguillo and Blas 2009). *Physalis gracilis* is a perennial herb, slightly erect to prostrate, profusely branched at the stem base, with branches reaching up to 90 cm (occasionally 1.5 m) in length and long internodes (> 6 cm). Flowering occurs throughout the year with yellow corollas bearing five distinct dark spots. The fruit is a berry, orange in color, and 8–15 mm in diameter. This species is distributed from Nayarit and San Luis Potosí, Mexico, through Central America to Panama, at elevations between 1,400 and 2,250 m asl (Martínez et al. 2023). It inhabits open areas within pine–oak and cloud forests and occurs as a weed in agricultural fields associated with these ecosystems (Martínez et al. 2023). Given its perennial nature and fruit sweetness, it is important to evaluate its performance in cultivation and yield potential as a possible horticultural crop. The reproductive system of *P. gracilis* remains unknown determining this is crucial for establishing its cultivation as a new crop. Therefore, this study aimed to determine the reproductive system of *Physalis gracilis* and evaluate its agronomic performance. Given that most *Physalis* species reproduce by cross-pollination, we hypothesized that *P. gracilis* is self-incompatible and will perform favorably under open-field cultivation.

## Materials and methods

### Study area and plant material

The study was conducted in the experimental fields, both open-field and greenhouse, of the University Center for Biological and Agricultural Sciences (CUCBA), University of Guadalajara, located in Zapopan, Jalisco, Mexico (20°44’41’’N, 103°30’58’’W, 1640 m asl). The site has Eutric Regosol soils with an average pH of 5.38 and a semi-warm temperate climate with warm and rainy summers (García 2004). The mean annual temperature is 19.6 °C, the mean annual precipitation is 979.6 mm, and the average relative humidity is 60% (García 2004). Accessions of *P. gracilis* were collected in the states of San Luis Potosí (codes 512 and 513) and Querétaro (code 432) in Mexico (Table 1).

**Table 1:** Geographical data of the studied populations of *Physalis gracilis*

Code	Acession*	State	Municipality	Locality	Elevation m asl
432	OVP 432	Querétaro	Pinal de Amoles	Pinal de Amoles	2340
512	LEV sn	San Luis Potosí	Xilitla	Xilitla	638
513	LEV sn	San Luis Potosí	Huehuetlán	Rancho la Pimienta	161

*LEV **=** Valdivia-Mares et al. 2015, OVP= Vargas Ponce et al. this study

### Reproductive system

To characterize the reproductive system, seeds from the three accessions were germinated in trays in a greenhouse using a substrate mixture of peat moss, regolith, and humus (3:2:1). This substrate provides the drainage, aeration, and moisture retention necessary for germination and seedling growth. When the seedlings reached 10 cm in height, they were transplanted into 10 cm diameter pots. One month later, they were transferred to 15 cm diameter pots containing a substrate composed of volcanic rock (pumice) and organic matter (soil) (1:1). Fifteen plants were established (5 plants per accession) in the greenhouse and other fifteen plants in open field, to evaluate the artificial crossing experiments and natural pollination by duplicating in each plant (30 replicates per treatment described in Sect.  2.4).

### Floral development and longevity

Floral longevity and developmental stages were assessed by monitoring flower buds from emergence to senescence in potted plants grown in a greenhouse and open field during the spring-summer cycle of 2023. Thirty buds were randomly selected (15 in the greenhouse and 15 in the open-field, one bud per plant), and observations to record different stages of floral development were made hourly from 9:00 to 17:00 h for seven days. In other thirty flowers in anthesis, we determined the timing of stigma receptivity and anther dehiscence. Each day after anthesis, the pistils were immersed in hydrogen peroxide to assess stigma receptivity. A stigma was considered receptive when a strong bubbling reaction was observed (Zeisler 1933).

### Artificial crossing experiments

The reproductive system of *Physalis gracilis* was determined using seven artificial crossing treatments, following the approach proposed by Solís-Montero et al. (2021). Five treatments were conducted under greenhouse, and two were conducted under open-field conditions. Artificial crosses were performed once the stigma receptivity was established. Experiments were conducted on 15 plants, with two replicates per treatment (30 flowers per treatment).

The five greenhouse artificial crossing treatments were as follows: (1) Agamospermy (A): This treatment evaluated the capacity of *P. gracilis* to produce fruit through endogenous mechanisms in the absence of pollen. Anthers were removed (emasculated with metal forceps) on the first day of anthesis, prior to anther dehiscence, to prevent self-pollen deposition. (2) Autonomous self-pollination (AS): Flowers were left intact and unmanipulated until senescence to determine whether *P. gracilis* is capable of autonomous self-pollination in the absence of pollinators. (3) Hand-mediated self-pollination (MS): This treatment was used to test for the presence of a self-incompatibility system by transferring pollen from a flower’s own anthers onto its stigma using metal forceps. (4) Hand-mediated cross-pollination with a single pollen donor (CP1): This treatment assessed whether fruit set could occur using pollen from another plant. Pollen from one flower of different individual was deposited onto the stigma of a flower that had been previously emasculated. (5) Hand-mediated cross-pollination with three pollen donors (CP3): To evaluate fruit set under excess pollen availability, anthers from three flowers (each from a different individual) were collected, placed in a 0.2 mL tube, mixed, and deposited onto the stigma of a previously emasculated flower using metal forceps. The two open-field treatments were as follows: (6) Pollen supplementation (PS): This treatment was used to determine whether plants were pollen-limited. Pollen from three different plants was mixed (one anther per flower in a 0.2 mL tube) and applied to the stigma of the selected flower. Following manual cross-pollination, the plants were exposed to open (natural) pollination. (7) Natural pollination (NP): Flowers were marked at the pedicel and left exposed to natural pollination. This treatment tested the capacity of *P. gracilis* to set fruit under natural conditions. For all treatments, the numbers of immature fruits (one week later) and mature fruits (approximately three weeks later) were recorded.

The self-incompatibility index (SII) was calculated as the ratio of mature fruits produced per flower in self-pollination treatments to that in cross-pollination treatments with a single pollen donor (Ruíz-Zapata and Arroyo 1978). The SII values were classified as follows: 1 = fully compatible; 0.2 > SII < 1 = partially compatible; SII < 0.2 = highly incompatible; and SII = 0 = completely incompatible. To evaluate pollen limitation, the pollen limitation index (PL) was calculated as PL = 1 - (NP/PS), where NP corresponds to immature fruit production under natural pollination and PS corresponds to that under pollen supplementation (Larson and Barrett 2000). Immature fruits were used to calculate PL index because some mature fruits were lost to pests. Negative or zero values indicated no pollen limitation, whereas positive values indicated pollen limitation (PL).

Additionally, the pre-dispersal fitness index was calculated as Wpre = 1-(WpreNP/WprePS), where WpreNP was obtained by multiplying fruit production by the mean number of seeds per plant under natural pollination, and WprePS was calculated similarly for the pollen-supplementation treatment (Gómez et al. 2010; Solís-Montero et al. 2015). Confidence intervals (95%) were estimated using bootstrapping with 1000 permutations (Gómez et al. 2010). Finally, differences in fruit set and seed number were analyzed using generalized linear models (GLM). A binomial distribution was used for the fruit set, and a Poisson distribution was used for the seed number. For fruit set, confidence intervals were estimated using the Hmisc library (Harrel and Dupont 2019). For seed number, post-hoc comparisons were performed using Tukey’s test with the glht function and the multcomp library (Hothorn et al. 2008). Treatments involving agamospermy, autonomous self-pollination, and hand-mediated self-pollination were excluded from the analysis as they did not produce fruit. All analyses, except for the SII self-incompatibility index, were performed using RStudio ver. 4.3.2 (R Core Team 2023).

### Agricultural management and agronomic performance

**Fig. 1:**
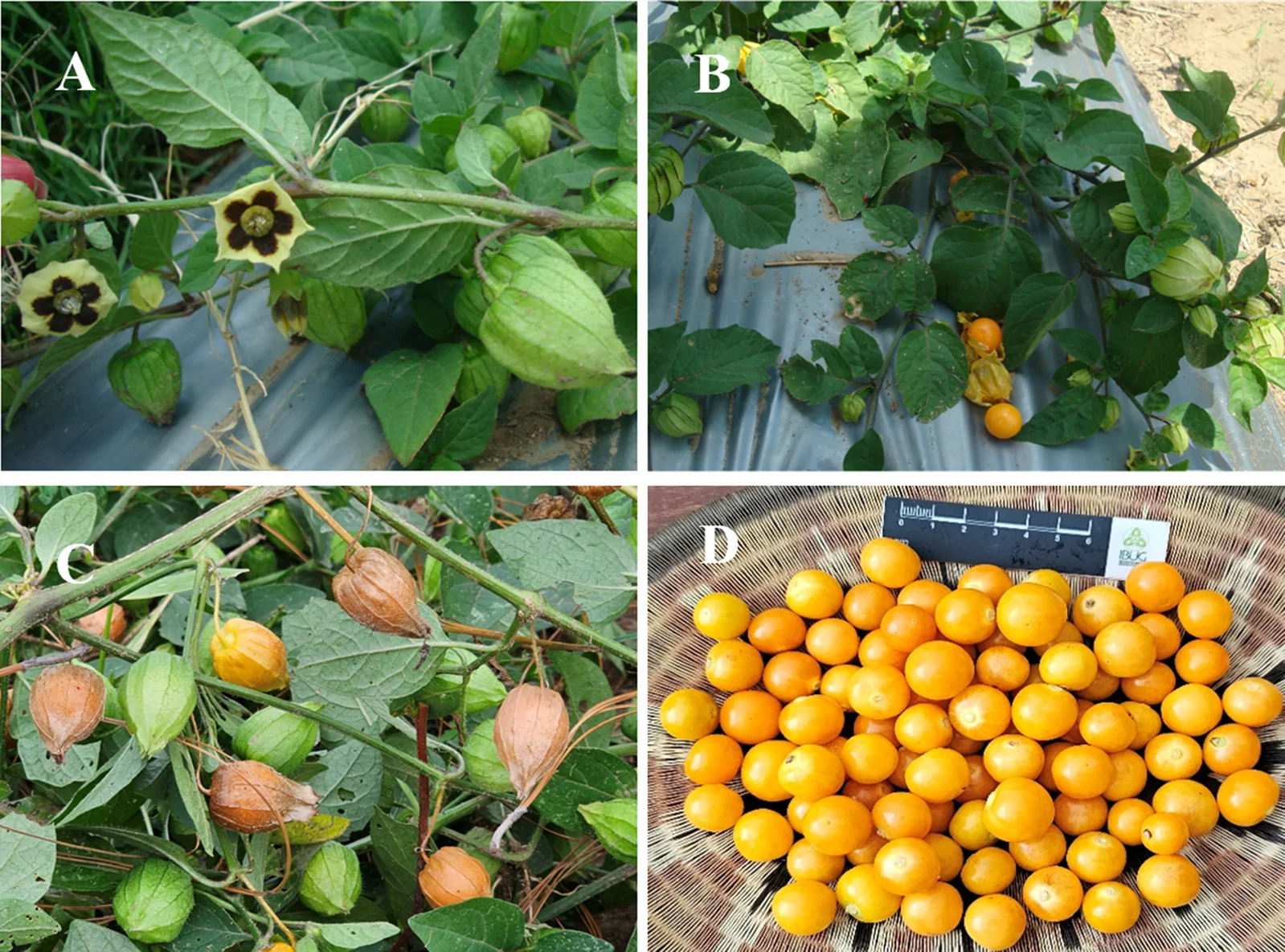
Cultivated Plants of *Physalis gracilis*. (**A**) flowers and rooting at the lower nodes, (**B**) prostrate habit, (**C**) mature fruits with husks, (**D**) fruits

Eighty seedlings of each of the three accession (10 cm in height) were transplanted in open fields in rows with mulch and drip irrigation (500 l·h⁻¹) (Fig. 1) at a spacing of 60 cm between plants to separate the individual plants and 150 cm between rows because *P*. *gracilis* develops long branches, in a continuous layout without replication, resulting in a planting density of 13,778 plants·ha⁻¹. Prior to planting, 500 kg of worm humus was incorporated into the experimental area to improve the soil quality. Agricultural management included weed control and the weekly application of fertilizer (Triple 20-20-20 NPK granular, at a dilution of 2 g/l, 1–4 kg/ha) throughout the experimental period. Organic insecticides were applied every 15 days, as needed. Data were collected from the fruits harvested from 48 plants of each accession.

Agronomic performance was evaluated using descriptors commonly applied to Mexican species of *Physalis*: total number of fruits per plant (NFP), average fruit weight (AFW), equatorial diameter of the fruit (DE), firmness (F), total soluble solids (TSS) expressed in Brix degrees, and pH (Table 2). Measurements were taken as reported by Morales et al. (2019) and Valdivia-Mares et al. (2016). Fruit weight was measured using an analytical balance (Nimbus^®^). The equatorial diameter was determined using a digital caliper (precision ± 0.01 mm), and fruit firmness was determined using an analog penetrometer (GY-3, resolution 0.02). TSS (°Brix) and fruit acidity (pH) were measured using a refractometer (Atago Ind) and digital potentiometer (Science Med, precision ± 0.05), respectively. The descriptors were measured in five randomly selected fruits per plant for each accession. For pH, the number of fruits varied for each plant depending on the juice availability. In addition, the yield per hectare (Yha) was calculated by multiplying the average values per individual plant of the number of fruits and fruit weight, as well as by the total planting density (i.e., 13,778 plants·ha⁻¹). This estimate generated only a single value per accession, which did not allow for statistical analysis.

**Table 2:** Descriptors used for the agronomic characterization of *Physalis gracilis*

1	Descriptor	Code	Type^a^	Units
	Total number of fruits per plant	NFP	A	amount
2	Average fruit weight	AFW	A	gr
3	Equatorial diameter of the fruit	ED	A	mm
4	Firmness	F	A	Kg/cm²
5	Total soluble solids	TSS	PCh	Brix grades
6	Hydrogen potential	pH	PCh	Log_n_ potential of hydrogen
7	Yield per hectare	Yha	A	t·ha^− 1^

^a^ A= agronomic, PCh = physicochemical

To evaluate the variation in descriptors among accessions, a prospective analysis was performed using a one-way permutational analysis of variance (ANOVA) for each descriptor among the accessions. A Euclidean distance matrix was constructed per each descriptor and the permutation ANOVA was performed according to the criteria of Anderson et al. (2008). Accessions were considered fixed effects, and statistical significance was assessed using 10,000 permutations based on the unrestricted permutation of raw data method and type III sums of squares. Pairwise comparisons were performed using Pseudo-t tests with adjusted p-values estimated by the Holm-Sidak correction. A test for homogeneity of multivariate dispersion (PERMDISP) was also used for univariate analysis, and behaved in the same way as Levene’s test of homoscedasticity. PERMDISP was applied to assess differences in the variability of descriptors among accessions using 10,000 permutations to assess statistical significance. The permutation ANOVA’s results were visualized using boxplots generated in Sigmaplot 11.0 (Systat Software, Inc.). Subsequently, the statistically different descriptors among accessions were analyzed using a one-way permutational multivariate analysis of variance (PERMANOVA) based on Euclidean distances and z-value normalization data pre-treatment. Statistical significance, pairwise comparisons, and PERMDISP were performed using the same criteria as those described for permutation ANOVAs. Finally, principal coordinate analysis (PCO) was performed to complement the PERMANOVA outputs, using the same data preprocessing and resemblance coefficient as PERMANOVA. PCO ordination was based on four descriptors (AWF, ED, F, and pH), as well as the yield per hectare (Yha), which were visualized through a Pearson multiple correlation showing the differences in agronomic attributes among accessions. All analyses were performed with Primer 6.1.11 + PERMANOVA 1.0.1 (Anderson et al. 2008).

## Results

### Reproductive system

#### Floral development and longevity

**Fig. 2:**
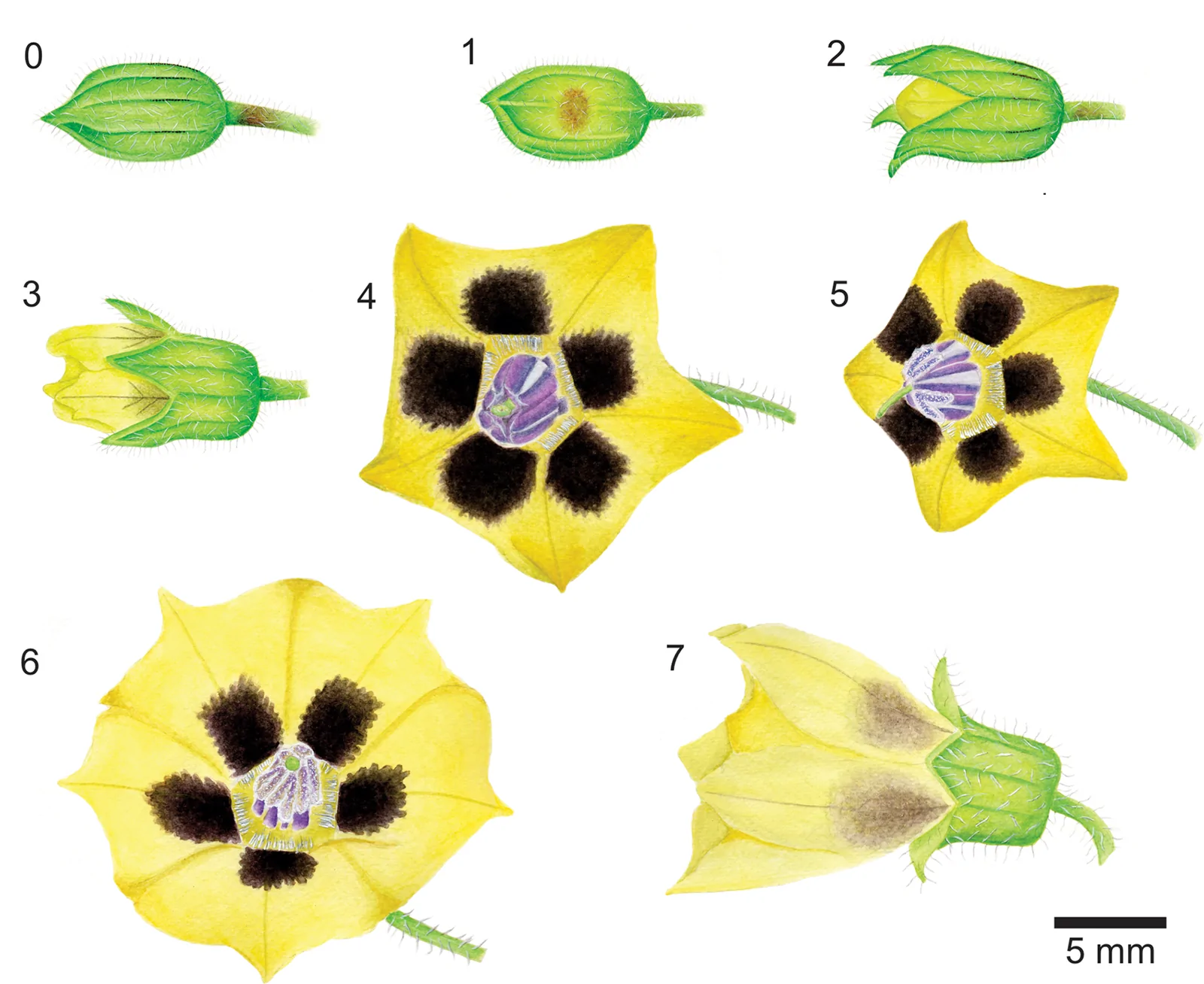
Floral developmental stages of *Physalis gracilis*. Codes: 0 = immature floral bud; 1 = mature floral bud (trichomes on the floral calyx); 2 = separation of the calyx lobes; 3 = beginning of corolla opening; 4 = full corolla opening; 5 = onset of dehiscence (in two anthers); 6 = full anther dehiscence; 7 = floral senescence. Scale = 5 mm

After monitored 28 flowers from bud to senescence in both environments (greenhouse and open-field), we defined eight developmental floral stages and longevity in *P. gracilis* (Fig. 2): 0) immature floral bud; (1) mature floral bud (with trichomes on the floral calyx); (2) separation of calyx lobes; (3) onset of corolla opening; (4) full corolla opening; (5) onset of anther dehiscence (two anthers); (6) full anther dehiscence; and (7) floral senescence. Flower longevity differed between environments: in the greenhouse, stages 3–7 lasted an average of four days, whereas in the open-field, they lasted an average of 2.5 days. *Physalis gracilis* flowers opening began between 9:00 and 10:00 a.m. and closing occurred around 5:00 p.m. Stigma receptivity was observed at stage 5, coinciding with the onset of anther dehiscence.

#### Artificial crosses

*Physalis gracilis* was confirmed as a self-incompatible species. The self-incompatibility index indicated a completely self-incompatible reproductive system (SII = 0). Neither agamospermy nor autonomous nor hand-mediated self-pollination resulted in fruit set. In contrast, treatments favoring cross-pollination (cross-pollination, pollen supplementation, and natural pollination) produced 84% of the fruits set to maturity. The statistical analysis (GLM) comparing the four treatments (CP1, CP3, PS, and NP) indicated significant differences; however, pairwise tests (Tukey) were not sufficiently robust to confirm these differences, likely due to the nature of the data (Table 3). Pollen supplementation (PS) appeared to generate lower fruit production than the other treatments, with only nine of the 30 expected fruits reaching maturity. Several fruits were lost in the open-field due to causes unrelated to the experiment. The pollen limitation (PL) and pre-dispersal fitness (Wpre) indices indicated no evidence of pollen limitation, as both values were negative, with PL = -0.17 ± 0.12 (upper and lower confidence intervals of -0.3927 and − 0.0127), and Wpre = -0.43 ± 0.12 (confidence intervals of -0.7184 and − 0.0910). Seed production varied among the different treatments. Crosses with a single donor (CP1) produced significantly fewer seeds than those with three donors (CP3), with the highest seed numbers observed under natural pollination (Table 3). Pollen supplementation with at least three donors yielded results comparable to those obtained with CP3.

**Table 3:** Immature fruit production (number of flowers per treatment) and seed number of mature fruits ± standard error (number of mature fruits) of *P. gracilis*

Treatment	GLM		
	Immature fruit production	Mean number of seeds	
Agamospermy	0 (30)	0	
Autonomous self-pollination	0 (30)	0	
Hand-mediated self-pollination	0 (30)	0	
Cross-pollination single pollen donor	100 (30)	188.43 ± 10.19 ^**c**^ (30)	
Cross-pollination three pollen donor	100 (30)	206.61 ± 9.18 ^**b**^ (28)	
Natural pollination	96.6 (30)	282.72 ± 13.39 ^a^ (29)	
Pollen supplementation	77.77(18)	205.11 ± 41.55 ^b^ (9)	
Deviance	12.654	533.94	
DF	3	3	
P	< 0.001	< 0.001	

Different letters indicate statistical differences between the treatments

*** *P* < 0.001

### Response to agricultural management

**Fig. 3:**
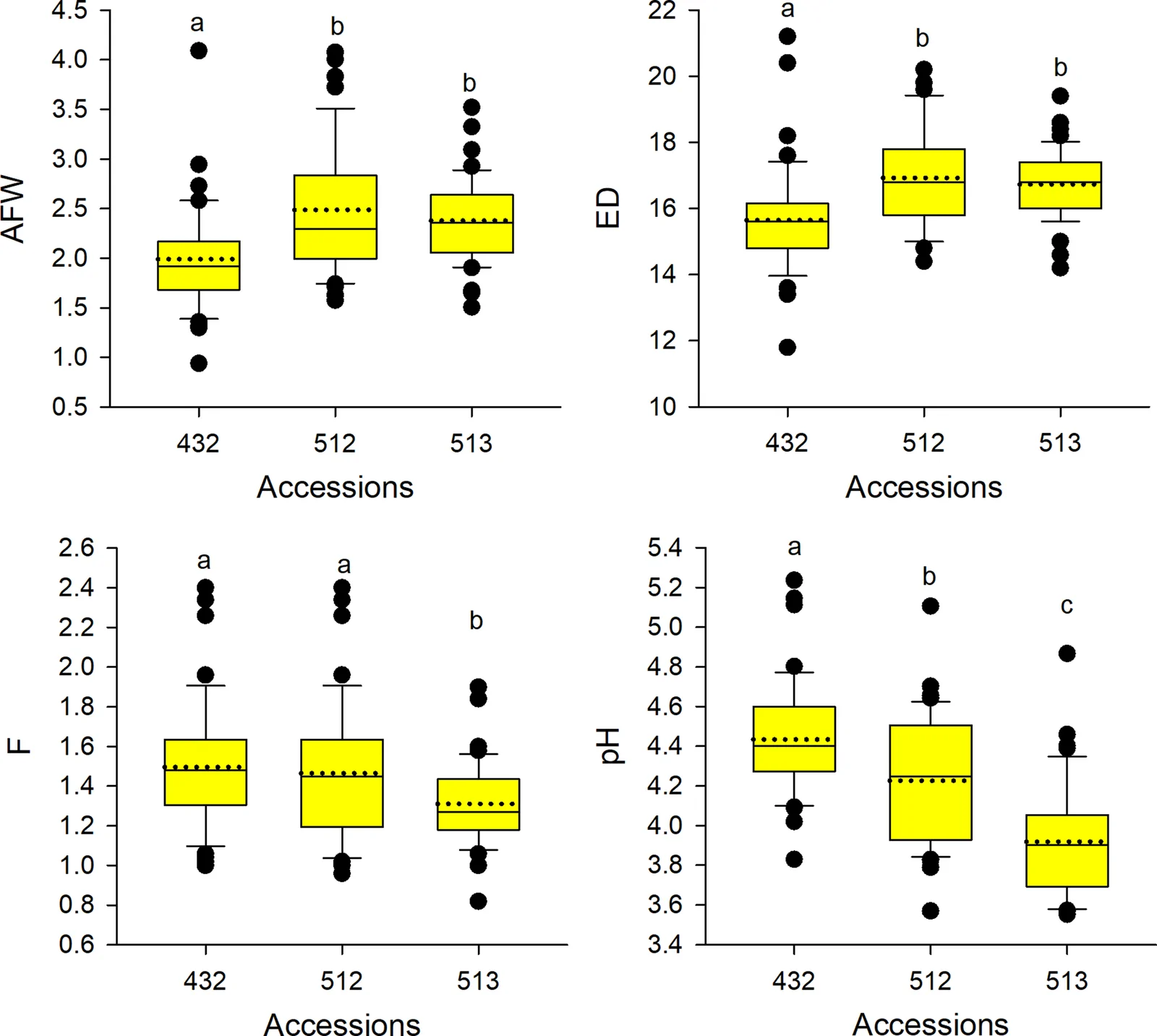
Boxplots showing the variation in significant agronomic descriptors among the *Physalis gracilis* accessions. Number of replicates (n) per accession = 48. Box plots show medians (solid line) or the 50th percentile (second quartile); the box (interquartile range [IQR]) indicates the 25th (first quartile) and 75th (third quartile) percentiles; whiskers represent the 5th and 95th percentiles; outliers are shown as small black circles, and the average corresponds to the dotted lines. Different letters indicate significant differences (*P* ≤ 0.05) among accessions, according to the results of the pairwise Pseudo-t test of the permutational ANOVAs. The p-values of pairwise comparisons were adjusted using the Holm-Sidak correction

A total of 162 plants from three *P. gracilis* accessions were established under agricultural management in open field. Their establishment and adaptation were favorable, achieving a survival rate of 93.8% and an average productivity of 8.178 t ha^− 1^ (Table 4). The one-way permutation ANOVAs results showed that most agronomic descriptors (AFW, ED, F, pH, Yha) were statistically different among the accessions studied, except for the number of fruits per plant (NPF) and the total soluble solids (TSS) (Table 5). These ANOVA models explained the total variation ranging from C.V. = 23.4% to C.V. = 46.4%. PERMDISP outputs showed that there was mostly a location effect (i.e., homogeneity of dispersions) in the descriptors with significant differences, except for average fruit weight (AFW), which had a dispersion effect (Table 5). The average number of fruits per plant (NPF) did not vary significantly among accessions, ranging from 247 in accession 512 to 278 in accession 513 (Table 4). In contrast, the average fruit weight (AFW) ranged significantly from 1.99 g to 2.48 g, with accessions 512 and 513 presenting the highest weights, while accession 432 had the lowest (Fig. 3; Table 4). The equatorial diameter (ED) differed significantly among accessions, with the highest values observed in accessions 513 (16.7 mm) and 512 (16.9 mm), and the lowest value (15.7 mm) was observed in accession 432 (Fig. 3; Table 4). Fruit firmness (F) was similar between accessions 432 (1.49 kg/cm ²) and 512 (1.46 kg/cm ²), both significantly exceeding that of 513 (1.31 kg/cm ²) (Fig. 3; Table 4). The total soluble solids (TSS) did not significantly change among accessions, fluctuating from 11.17 to 11.36 °Brix (Table 4). However, the hydrogen potential (pH) showed higher acidity in accession 513 (3.91) and lower acidity in accession 432 (4.43) (Fig. 3; Table 4). The estimated yield per hectare (Yha) differed among the accessions. Accession 513 showed the highest values of Yha = 9.07 t·ha^− 1^, as well as the highest proportion of non-established plants (9.2%), whereas accession 432 recorded the lowest yield (6.99 t·ha^− 1^) (Table 4).

**Table 4:** Means and standard deviations per accession of descriptors used in *Physalis gracilis*

Accession		NPF amount	AFW gr	ED mm	F Kg/cm²	TSS Brix grades	pH Log_*n*_ potential of hydrogen	Yha t·ha^− 1^
**432**		255	1.99	15.65	1.49	11.34	4.43	6.991
	SD	± 103	± 0.51	± 1.60	± 0.31	± 2.17	± 0.28	
	minimum	59	0.93	11.8	1	5.32	3.83	
	maximum	504	4.09	21.2	2.4	15.44	5.23	
**512**		247	2.48	16.92	1.46	11.17	4.22	8.439
	SD	± 45.36	± 0.64	± 1.49	± 0.33	± 1.84	± 0.35	
	minimum	12	1.57	14.4	0.96	6.08	3.57	
	maximum	626	4.07	20.2	2.4	14.08	5.10	
**513**		278	2.37	16.72	1.31	11.36	3.91	9.077
	SD	± 159.08	± 0.42	± 1.01	± 0.20	± 1.78	± 0.27	
	minimum	46	1.50	16	0.82	7.16	3.55	
	maximum	744	3.52	19.4	1.9	14.72	4.86	
	M	**260.36**	**2.28**	**16.43**	**1.42**	**11.29**	**4.18**	**8.178**

NPF = total number of fruits per plant, AFW = average fruit weight, ED = equatorial diameter of the fruit, F = firmness, Yha = yield per hectare (t-ha^− 1^), SD = standard deviation, M = mean of the three accessions

**Table 5:** Results of one-way permutational ANOVA and PERMANOVA, such as PERMDISP outputs of the descriptors’ variation among the *Physalis gracilis* accessions

Source of variation	Permutational ANOVA / PERMANOVA			PERMDISP		Pairwise comparisons			
	PSeudo-F	*P*	C.V. (%)	F	*P*	Groups	t	*P*	*P*_adjusted_
NFP									
Accessions	0.6304	0.5311	8.1	4.2191	**0.0196**				
Residuals			91.9						
AFW									
Accessions	11.547	**0.0002**	31.9	5.2506	**0.0121**	432, 512	4.1988	**0.0001**	***
Residuals			68.1			432, 513	4.0545	**0.0001**	***
						512, 513	0.99198	0.3172	ns
ED									
Accessions	11.612	**0.0001**	32.0	2.6441	0.0874	432, 512	4.0193	**0.0003**	***
Residuals			68.0			432, 513	3.9424	**0.0001**	***
						512, 513	0.737	0.4734	ns
F									
Accessions	5.4605	**0.0048**	23.4	3.4253	0.0503	432, 512	0.4578	0.6525	***
Residuals			76.6			432, 513	3.3746	**0.001**	***
						512, 513	2.6943	**0.0083**	ns
TSS									
Accesssions	0.1343	0.8717	11.8	0.8332	0.4976				
Residuals			88.2						
pH									
Accessions	37.109	**0.0001**	46.4	0.8535	0.4441	432, 512	3.3685	**0.0007**	***
Residuals			53.6			432, 513	8.887	**0.0001**	***
						512, 513	5.0566	**0.0001**	***
Significant agronomic descriptors									
Accessions	14.926	**0.0001**	35.0	6.921	**0.0023**	432, 512	3.3132	**0.0007**	***
Residuals			65.0			432, 513	5.3095	**0.0001**	***
						512, 513	2.7993	**0.0003**	***

P = permutational p-value (*P* ≤ 0.05), PAdjusted = p-value adjusted by the Holm-Sidak correction, C.V. (%) = coefficient of variation represented as a percentage, F = F-Fisher, t = Pseudo-t, NFP = total number of fruits per plant, AWF = average fruit weight, ED = equatorial diameter of the fruit, F = firmness, TSS = total soluble solids (°Brix), pH = hydrogen potential

### Agronomic performance

**Fig. 4:**
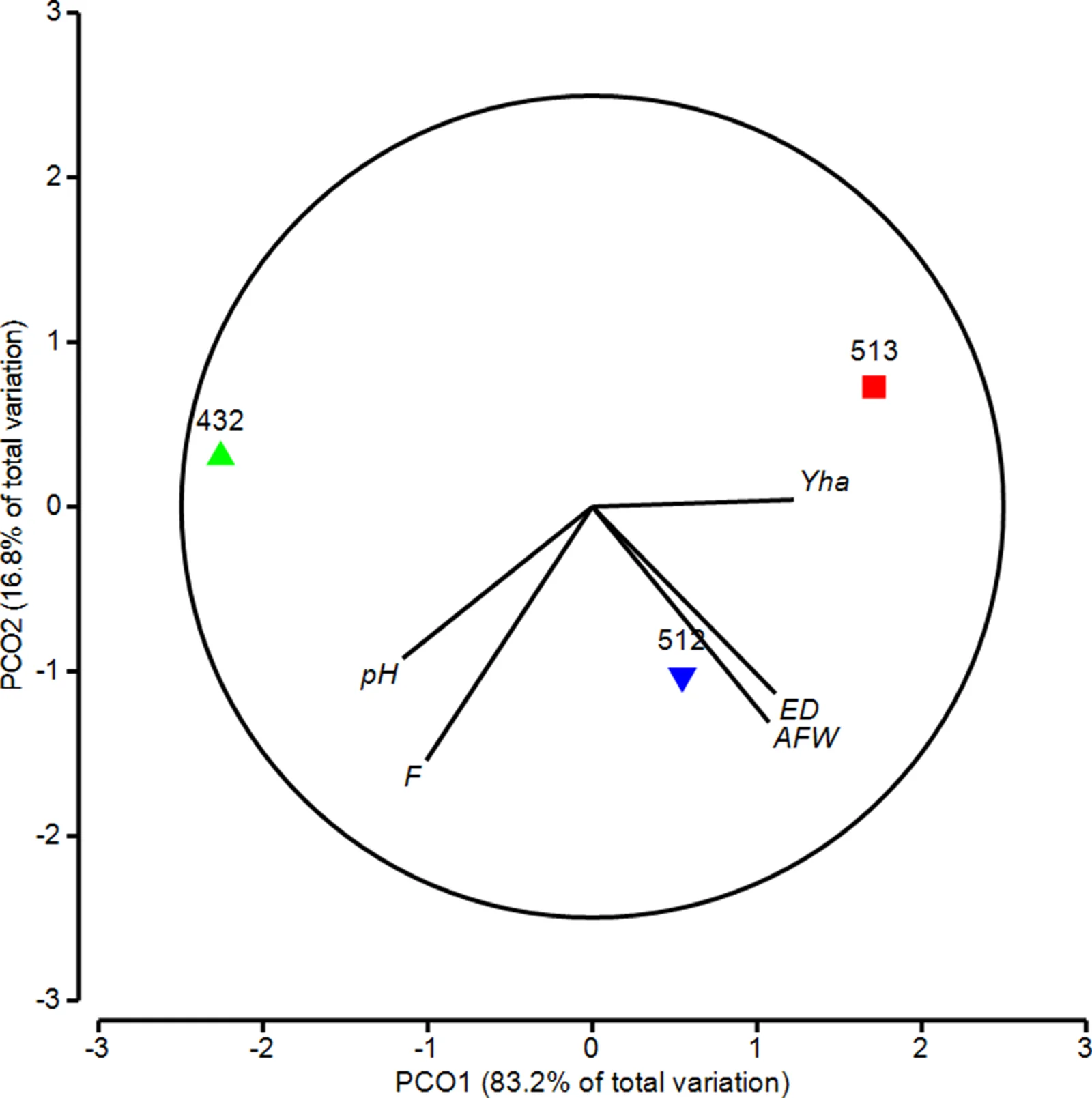
Principal Coordinates Analysis (PCO) ordination based on the mean values of the four significant agronomic descriptors from three accessions of *Physalis gracilis*. Codes: 432 = accession from Pinal de Amoles, Queretaro, 512 = accession from Xilitla, San Luis Potosi, 513 = accession from Huehutlan, San Luis Potosí, TSS = Total soluble solids, NFP =Total number of fruits per plant, DE = Equatorial diameter of the fruit, AFW= Average fruit weight, F = Firmness, pH = Hydrogen potential

The one-way PERMANOVA model had an explained variation of C.V. = 35.0% of the agronomic descriptors among the *Physalis gracilis* accessions, revealing statistically significant differences (Table 5). Pairwise comparisons confirmed significant differences in the traits AFW, ED, F and PH, particularly in the comparisons of accession 432 with accessions 512 and 513 (Table 5). PERMDISP further indicated significant differences in the dispersion of descriptors among accessions, suggesting that both dispersion and location effects contributed to this variation (Table 5). The principal coordinate analysis (PCO) ordination performed at the accession level based on the mean values of descriptors showed that the first axis explained 83.2% of the total variation, which was primarily associated with yield per hectare (Yha) and pH (Fig. 4). Along this axis, accession 513 was strongly associated with Yha, whereas 432 was mainly related to pH, as well as with firmness (F). The second axis accounted for 16.8% of the variation and was mainly correlated with F, equatorial diameter (ED), and average fruit weight (AFW). Accession 512 was closely associated with ED and AFW compared to other accessions (Fig. 4).

## Discussion

Outcrossing plants are generally expected to experience stronger pollen limitations because they rely on pollinators for reproduction (Larson and Barret 2000). Contrary to this expectation, *Physalis gracilis* did not exhibit pollen limitation, despite being a completely self-incompatible species. Moreover, the high efficiency of its pollination is reflected in the evaluated agronomic traits, underscoring its potential as a monoculture crop.

### Reproductive system

#### Floral development and longevity

The floral anthesis of *P. gracilis* differed between individuals grown under open-field and greenhouse conditions. In the greenhouse, anthesis lasted almost twice as long, averaging four days and up to seven days for some plants. This extended duration is likely due to the absence of natural pollination, in contrast to open fields, where pollinators are present. For *P. angulata*, a self-compatible species, Figueiredo et al. (2020) reported a shorter anthesis period of two days, consistent with what we observed for *P. gracilis* under open-field conditions. Similarly, Lagos et al. (2008) found that in *P. peruviana*, a species with a mixed mating system (Ramírez et al. 2021), anthesis lasted only for two days. Recently, Ojeda-Flores (2024) reported that *P. cinerascens* (self-incompatible) and *P. nicandroides* (self-compatible) differ in their floral longevity, with a longer anthesis in *P. cinerascens* than in *P. nicandroides*. These findings align with our observations in *P. gracilis* under greenhouse conditions and are consistent with the general expectation that self-incompatibility reproductive systems prolong anthesis to increase pollination opportunities (Wyatt 1981; Gibbs and Talavera 2001; Weber and Goodwillie 2013 ; Ortiz et al. 2006; Martén-Rodríguez and Fenster 2008). The short duration observed in open-field *P. gracilis* plants is ecologically meaningful because floral anthesis influences the probability and frequency of pollinator visitation (Primack 1985). Ashman ( 2004) suggests that floral longevity is “a trait shaped by natural selection to suit a plant’s ecological context.” Studies on diverse plant groups support this view (Stead and Moore 1983; Ashman and Schoen 1997; Clark and Husband 2007; Castro et al. 2008; Prokop et al. 2021; Prokop 2024). Recently, Prokop (2024) found that in *Convolvulus arvensis*, a completely self-incompatible species, floral longevity varies with pollinator abundance, and that reductions in anthesis duration depend on the presence of pollinator species.

In our study, natural pollination resulted in a very high fruit set (96.6%), suggesting that both pollinator abundance in the open field and prolonged floral longevity in the absence of pollinators under greenhouse conditions are important. Although *Physalis* species differ in life cycles and reproductive systems (e.g., *P. gracilis* is perennial and self-incompatible; *P. angulata* is an annual self-compatible; *P. peruviana* is perennial with a mixed system), anthesis duration among them is surprisingly similar. This pattern contrasts with the expectation that reproductive systems should drive substantial differences in floral longevity (Ashman et al.^ 2004^).

#### Artificial crosses

The absence of fruit development, both autonomous and hand-mediated self-pollination, confirms that *P. gracilis* has genetic mechanisms that recognize and reject its self-pollen (Takamaya and Isogai 2005). In Solanaceae, approximately 39% of species have a gametophytic self-incompatibility (GSI) system mediated by RNases, which recognize and reject self-pollen grains to prevent self-fertilization (Franklin-Tong and Franklin 2003; Igic et al. 2006). Our results strongly suggest that *P. gracilis* has a self-incompatibility system that promotes outcrossing; however, future studies are needed to confirm that it is a GSI. Moreover, the agamospermy treatment indicated that the absence of endogenous mechanisms enabling fruit development without pollination does not occur.

#### Pollen limitation

Our results confirmed that *P. gracilis* is a completely self-incompatible species. Self-incompatibility has been reported in *P. viscosa* (Sullivan 1985), populations of *P. peruviana* (Ramírez et al. 2021), and cultivated populations of *P. philadelphica* (Solís-Montero et al. 2021). Although *P. acutifolia* is considered self-incompatible within populations breakdowns of this system have been observed (Pretzs and Smith 2021), as has recently been reported in *P. cinerascens* (Ojeda-Flores 2024). Self-incompatibility is thought to represent the ancestral state of *Physalis* (Igic et al. 2006), yet only *P. viscosa* had previously been described as completely self-incompatible (Sullivan 1985; Figueiredo 2020), to which *P. gracilis* can now be added.

According to the pollen limitation hypothesis, cross-pollinated species are expected to experience stronger limitations owing to pollen availability or pollinators (Larson and Barret 2000). Contrary to this expectation, *P. gracilis* exhibited no pollen limitation, and both fruit and seed production were higher under natural pollination than under pollen supplementation, suggesting that pollinators ensured sufficient pollen transfer to the flowers. This fruit and seed production indicates highly efficient plant-pollinator interactions. The reduction in seed production observed under pollen supplementation may be explained by competition among growing pollen tubes. Specifically, the deposition of excessive pollen grains on the stigma may cause saturation, where only a fraction of the pollen tubes successfully fertilize the ovules (Mulcahy and Mulcahy 1987).

Although floral visitors were not systematically quantified, field observations indicated frequent visits by *Apis mellifera.* Related to this, Delmas et al. ( 2015) found that in *Rhododendron ferrugineum*, pollen limitation increases with population density due to visitation rates per flower. In contrast, smaller populations may experience less pollen limitation but can also be less attractive to pollinators (Ashman et al. 2004). In this study, the experimental pot was small but was surrounded by diverse crops, which may have enhanced the pollinator attraction.

Seed production varied among the cross-pollination treatments. Pollination with a single donor or three individuals (including supplementation treatment) produced fewer seeds than natural pollination, which yielded the highest values. This intriguing result is probably attributed to the fact that in cross-pollination treatments (with one or three donors), we did not place enough pollen to fertilize many ovules, similar to pollinators. In contrast, when the stigma is saturated with enough pollen and receives more visits, pollen deposition decreases (Solís-Montero and Vallejo-Marín 2017). In the pollen supplementation treatment, we added extra pollen to flowers exposed to natural pollination and then removed pollen previously deposited by pollinators. Future studies are required to corroborate these hypotheses.

### Agricultural management and agronomic performance

*Physalis gracilis* showed successful establishment rate of 93.8% under cultivation. The yield obtained (7–9 t·ha^− 1^) fell within the range recorded for *P. pubescens* (7.5–12.5 t·ha^− 1^) and *P. acutifolia* (4.9–20.8 t·ha^− 1^), but was lower than that of *P. chenopodiifolia* (16–20 t·ha^− 1^) (Valdivia-Mares et al. 2016). Likewise, the yield per hectare of *P. gracilis* is modest when compared to the most widely cultivated species, *P. philadelphica* (0.63-20 and up to 30 t·ha^− 1^) (Santiaguillo et al. 1998) and *P. angulata* (8 and up to 28 t·ha^− 1^, Morales-Saavedra et al. 2019). It has also been reported that ground-level cultivation systems generally yield less than staked systems (Castillo et al. 1992).

In Mexico, *Physalis* species are typically grown as monocultures on the ground without a staking system. Morphological traits in cultivated annual species (*P*. *philadelphica* and *P. angulata*), such as erect growth, dichotomous branching, and short internodes, contribute to high fruit production and higher yields. These traits contrast with the attributes of *P. gracilis*, which is a perennial herbaceous plant with a decumbent to slightly erect growth habit, often reclining over neighboring species in its natural habitat. However, because of the several branches that grow from their stem base, the number of fruits produced per plant is similar to that of annual species. This prostrate growth appears to be less advantageous for cultivation; therefore, implementing staking or similar systems could enhance production. Notably, for *P. peruviana*, a perennial species with erect growth and profuse branching, X- and V-type staking systems have been successfully applied (Fischer et al. 2005).

The agronomic performance of *P. gracilis* is noteworthy for its possible establishment as a new crop, particularly because of its desirable fruit characteristics. Fruit weight (1.99–2.48 g) and equatorial diameter (15.65–16.92 mm) were higher than those reported by Valdivia-Mares et al. (2016) for the three wild species studied (*P. acutifolia*, *P. pubescens*, *P. chenopodiifolia*), and even higher than cultivated *P. angulata* (1.64 g and 12.63 mm). Likewise, sweetness, expressed as total soluble solids (11.17–11.36 ºBrix), was superior to that of the afore mentioned wild species (7.43–10.88 ºBrix), which are typically acidic or only slightly sweet, such as *P. chenopodiifolia*. The sweetness of *P. gracilis* fruits is comparable to values reported for *P. peruviana* (10.86–15.5 ºBrix), as are the pH values (3.88 in *P. peruviana*; 4.18 in *P. gracilis*) (Novoa et al. 2006; Madruga et al. 2009; Puente et al. 2011; Ramadan 2011).

*Physalis* fruits are rich in minerals, vitamins, polyphenols, and carotenoids—bioactive compounds that offer health benefits due to their antioxidant capacity and nutritional value (Ramadan 2011; Puente et al. 2011; Naranjo-Durán et al. 2023). Therefore, the orange-colored fruits of *P. gracilis* represent a potential alternative as a valuable source of nutrients and carotenoids, which have not yet been characterized. Moreover, their potential versatility, whether consumed fresh, as fruit, juice, jam, or dried, enhances their attractiveness.

PERMANOVA revealed significant differences among the three *P. gracilis* accessions analyzed, and PCO helped visualize the relationship among agronomic variables and each accession. The San Luis Potosí accessions (512 and 513) were found to be the most promising ones. Accession 512 showed superior traits for selecting plants with larger and heavier fruits, whereas 513 presented the highest number of fruits per plant and the best ºBrix value. Accession 432 produced smaller fruits with high sweetness levels. Interestingly, accessions 513 and 432 combined high sweetness values with small fruit size, indicating a negative correlation between fruit size and sweetness, a trend also observed in wild accessions of *Solanum lycopersicum*, which poses challenges for breeding programs (Luengwilai et al. 2010). The variation observed among the *P. gracilis* accessions was consistent with the morphological variability expected in cross-pollinated species (Olsen and Wendel 2013).

### Reproductive system: advantages and limitations for cultivation

*Physalis gracilis* is completely self-incompatible; it relies entirely on cross-pollination for reproduction. This condition could initially represent a disadvantage in achieving phenotypic uniformity and establishing the species as monocultures. However, our results demonstrate that pollinators ensure high fruit and seed production, suggesting that cross-pollination may also confer advantages, particularly in terms of crop resistance to pests, diseases, and herbivory. For instance, Ruiz-Arocho et al. (2024) reported that herbivory was lower in cultivated populations of *P. philadelphica* than in wild populations, possibly because of the greater genetic diversity generated by cross-pollination during domestication of the former.

Although approximately 25% of cultivated species are self-incompatible, only a minority are completely self-incompatible, whereas most exhibit partial self-incompatibility (Dempewolf et al. 2012). Furthermore, some self-incompatible species subjected to artificial selection tend to undergo transitions toward self-compatibility, thereby facilitating the fixation of desirable traits (Rick 1988a, b). In this regard, *P. gracilis* may not be exempt from changes in its reproductive system, especially since breakdowns of self-incompatibility have been documented in *P. acutifolia*, even within individual plants (Pretzs and Smith 2021).

Cross-pollination also offers advantages in terms of crop productivity (Rosas et al. 2010; Klein et al. 2018). Approximately 70% of self-incompatible crops exhibit higher yields when animal-mediated pollination occurs (Roubik, 1995; Willmer 2011). Additional benefits include increased seed production in alfalfa, improved fiber quality in cotton, and up to a 50% increase in raspberry fruit weight (Rhodes 2002; Willmer 2011). Therefore, quantifying the contribution and estimating the value of cross-pollination in *P. gracilis* is essential for assessing its potential as a monoculture.

## Conclusions

The assessment of the reproductive system of P. gracilis, based on a small sample size, showed the three evaluated accessions of *Physalis gracilis* exhibited a completely self-incompatible reproductive system that does not exhibit pollen limitation and responds positively to agricultural management. While self-incompatibility entails complete reliance on pollinators, in these accessions, it does not appear to be a limitation or disadvantage, as efficient pollination ensures consistent fruit production. Future studies could expand the sampling to include a larger number of populations and individuals and provide further evidence regarding the reproductive system of the species. Although the per-hectare productivity of *P. gracilis* is comparable to that of other *Physalis* species, it is relatively low compared to that of the most widely cultivated species, but posible interesting for local communities. Nevertheless, agronomic practices such as staking or trellising can improve productivity. Importantly, *P. gracilis’s* favorable fruit quality traits, particularly sweetness (11.17–11.36 ºBrix), comparable to that of cape gooseberry (*P. peruviana*), a fruit highly valued in South America, highlight its potential as a promising agro-food resource.

## Data Availability

Not applicable.
